# Web-Based Lifestyle Interventions for Prostate Cancer Survivors: Qualitative Study

**DOI:** 10.2196/19362

**Published:** 2020-11-10

**Authors:** Elizabeth Y Wang, Rebecca E Graff, June M Chan, Crystal S Langlais, Jeanette M Broering, Justin W Ramsdill, Elizabeth R Kessler, Kerri M Winters-Stone, Erin L Van Blarigan, Stacey A Kenfield

**Affiliations:** 1 University of California, San Francisco San Francisco, CA United States; 2 Columbia University Vagelos College of Physicians and Surgeons New York, NY United States; 3 Oregon Health and Sciences University Portland, OR United States; 4 University of Colorado Cancer Center Aurora, CO United States

**Keywords:** cancer survivorship, digital health, technology-based intervention, internet-based intervention, usability

## Abstract

**Background:**

Exercise and a healthy diet can improve the quality of life and prognosis of prostate cancer survivors, but there have been limited studies on the feasibility of web-based lifestyle interventions in this population.

**Objective:**

This study aims to develop a data-driven grounded theory of web-based engagement by prostate cancer survivors based on their experience in the Community of Wellness, a 12-week randomized clinical trial designed to support healthy diet and exercise habits.

**Methods:**

TrueNTH’s Community of Wellness was a four-arm pilot study of men with prostate cancer (N=202) who received progressive levels of behavioral support (level 1: website; level 2: website with individualized diet and exercise recommendations; level 3: website with individualized diet and exercise recommendations, Fitbit, and text messages; and level 4: website with individualized diet and exercise recommendations, Fitbit and text messages, and separate phone calls with an exercise trainer and a registered dietitian). The primary aim of the study is to determine the feasibility and estimate the effects on behaviors (results reported in a separate paper). Following the 12-week intervention, we invited participants to participate in 4 focus groups, one for each intervention level. In this report, we used grounded theory analyses including open, axial, and selective coding to generate codes and themes from the focus group transcripts. Categories were refined across levels using embodied categorization and constant comparative methods.

**Results:**

In total, 20 men with prostate cancer participated in the focus groups: 5, 4, 5, and 6 men in levels 1, 2, 3, and 4, respectively. Participants converged on 5 common factors influencing engagement with the intervention: environment (home environment, competing priorities, and other lifestyle programs), motivation (accountability and discordance experienced within the health care system), preparedness (technology literacy, health literacy, trust, and readiness to change), program design (communication, materials, and customization), and program support (education, ally, and community). Each of these factors influenced the survivors’ long-term impressions and habits. We proposed a grounded theory associating these constructs to describe the components contributing to the intuitiveness of a web-based lifestyle intervention.

**Conclusions:**

These analyses suggest that web-based lifestyle interventions are more intuitive when we optimize participants’ technology and health literacy; tailor interface design, content, and feedback; and leverage key motivators (ie, health care providers, family members, web-based coach) and environmental factors (ie, familiarity with other lifestyle programs). Together, these grounded theory–based efforts may improve engagement with web-based interventions designed to support prostate cancer survivorship.

## Introduction

Prostate cancer is the most common cancer among men in the United States, with more than 190,000 new diagnoses expected in 2020 [1]. The median age at diagnosis is 66 years, and 82% of men are aged 65 years or older [2]. Many men live for decades after their diagnosis and may benefit from adopting healthy dietary and exercise practices to combat prostate cancer symptoms and treatment-related side effects [3-8] in addition to improving their overall health.

Diet and exercise are associated with lower risk of prostate cancer progression [9], prostate cancer–specific mortality [10-13], and treatment-related side effects [14-18]. Specifically, cruciferous vegetables, vegetable fat, fish, and cooked tomatoes [19] have been associated with lower risk of prostate cancer progression and/or mortality, whereas whole milk and poultry with skin have been associated with increased risk of prostate cancer progression and/or mortality [19-27]. Physical activity has also been consistently associated with significant reductions in mortality [26], symptoms, and treatment-related side effects. The 2018 American College of Sports Medicine roundtable recommendations for cancer survivors include 30 min of moderate aerobic training 3 or more times a week for at least 8 to 12 weeks; resistance training alone or the addition of resistance exercise to an aerobic regimen may also improve symptoms [28]. The Exercise and Sports Science Australia recommends that the specifics of the multimodal exercise prescription and total weekly dosage be determined by the patient’s needs or goals but similarly supports that cancer survivors should *avoid inactivity* [29]. Unfortunately, many prostate cancer survivors fail to meet physical activity or nutrition recommendations.

Web-based interventions have the potential as scalable modalities to deliver lifestyle interventions in prostate cancer survivors [30]. Previous studies have demonstrated the benefits of web-based interventions in supporting behavior change related to diet, exercise, and smoking cessation for noncancer populations [31-35]. However, there remains to be a lack of data on the specific types and quantities of intervention components needed to change behavior. Thus, we developed a trial [36] to assess the feasibility and acceptability of a web-based intervention for men with prostate cancer. The study focused on the diet and exercise factors mentioned earlier, with particular attention to whether progressive levels of support would lead to increasingly higher levels of behavioral change and improvements in other outcomes such as symptom reduction and quality of life. Our primary feasibility, acceptability, and behavior change results are presented elsewhere.

Given the success of recent web-based interventions, we were also interested in the insights underlying the behaviors of prostate cancer survivors navigating a web-based platform. The attitudes, motivations, and perspectives of cancer survivors engaging in a web-based lifestyle intervention are complex and require further study. A prior qualitative study exploring lifestyle change in prostate, colon, and breast cancer survivors after participation in web modules designed to promote physical activity and healthy eating examined barriers to behavior change (knowledge, motivation, and individual reactions to cancer diagnosis) using a thematic analysis approach [37]. However, qualitative evidence to specifically inform intervention design is lacking. To our knowledge, perceptions of web-based interventions for lifestyle change in prostate cancer survivors have not been investigated using a qualitative methodology; as such, a grounded theory qualitative investigation using data-driven analysis would be helpful to inform future web-based intervention design. In this report, we aim to explore the insights of prostate cancer survivors who engaged with a web-based lifestyle intervention and to provide grounded theory–based recommendations to guide future intervention design.

## Methods

### Design

**Figure 1:**
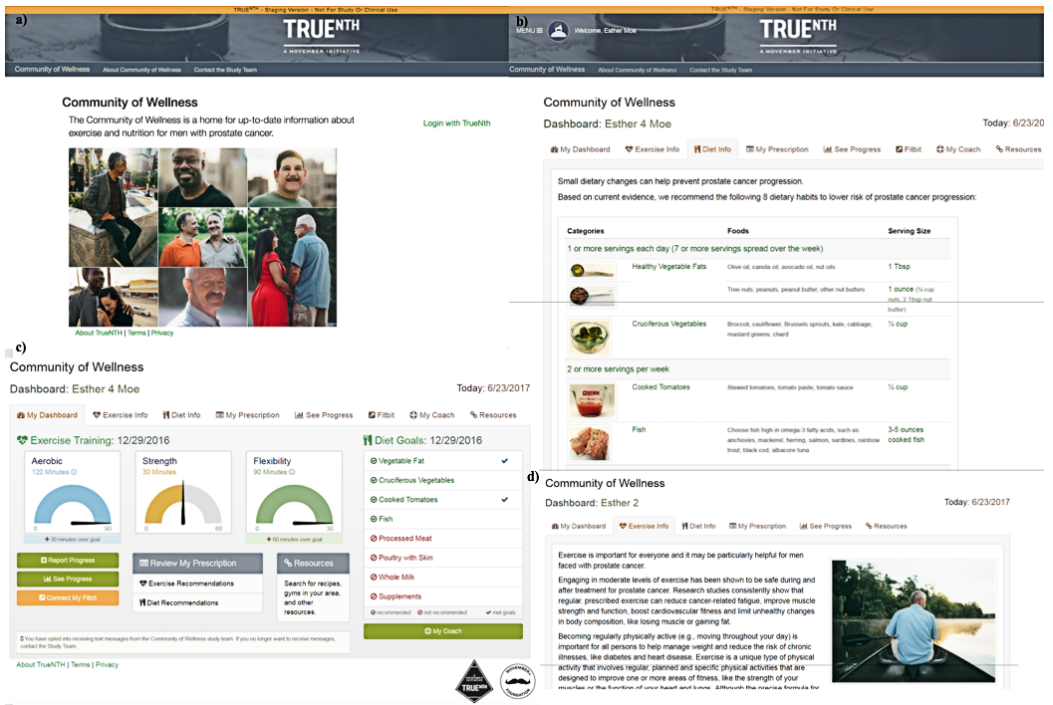
Screenshots from the Community of Wellness website (different view by level): (a) welcome page (levels 1-4), (b) diet information (levels 1-4), (c) dashboard (level 4), and (d) exercise information (levels 1-4).

We conducted a four-arm study called TrueNTH’s Community of Wellness (NCT03406013) of men with prostate cancer (N=202) who were randomized to receive progressive levels of behavioral support. The details of the design of the pilot study have been previously published [36], and select screenshots from the website are presented in Figure 1. Men in level 1 had access to prostate cancer–specific diet and exercise resources through a static, informational website. Men in level 2 had access to the website and received individualized diet and exercise recommendations based on a self-report survey completed at the start of the study. Men in level 3 had access to the website and received individualized diet and exercise recommendations and also received a Fitbit device and text messages. Men in level 4 had access to the website and received individualized diet and exercise recommendations, received a Fitbit device and text messages, and were offered a 30-min phone call with an exercise trainer and a 30-min phone call with a registered dietitian. Of note, the Community of Wellness is one of many TrueNTH programs funded by the Movember Foundation, and some men participated in multiple TrueNTH programs concurrently. Reporting in this study is consistent with the consolidated criteria for reporting qualitative research [38].

### Focus Groups

**Figure 2:**
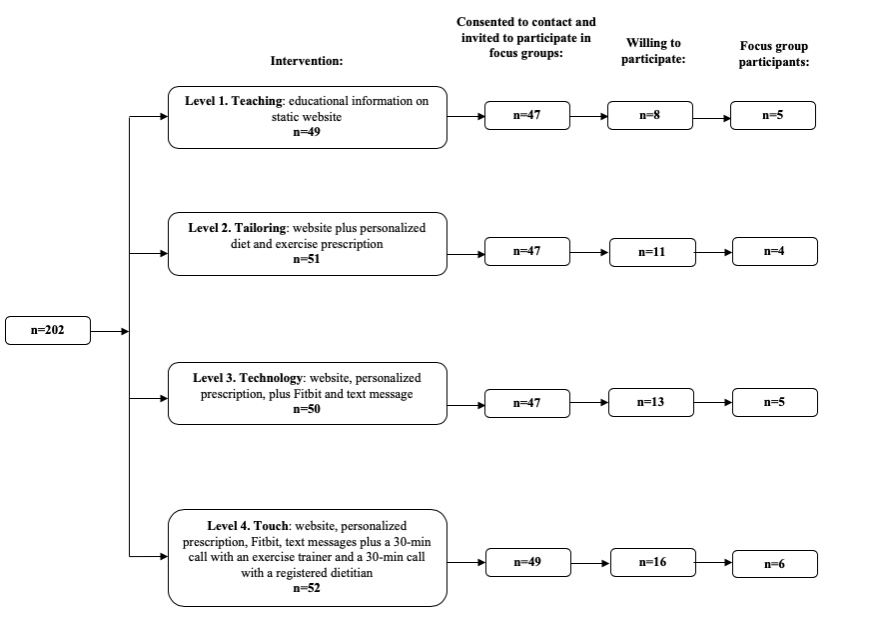
Community of Wellness study recruitment to intervention and focus groups.

Men who completed the pilot study and consented to being contacted were invited via email to participate in a focus group. Briefly, for the primary pilot trial, men were recruited through hospital cancer registry databases at the University of California San Francisco, the Oregon Health and Sciences University, and the University of Colorado Denver; at the Cancer of the Prostate Strategic Urologic Research Endeavor registry of men with prostate cancer; and in clinics at the abovementioned institutions. Each participant consented to participate in both the pilot study and focus group. In total, 48 men were willing to participate in a focus group; of these, 20 men could attend at the scheduled times (Figure 2). We conducted 4 focus groups, one for each intervention level. As participants in the trial could reside throughout the United States, focus groups were conducted via Zoom, a secure, interactive audioconference platform. In the interest of confidentiality, we disabled video calling; however, we used screen sharing so that participants could comment on various aspects of the website.

The focus groups were led by a female researcher (RG). She is an assistant professor in epidemiology with 10 years of experience researching urologic cancers, who previously worked in market research and web usability where she gained experience in qualitative research methods. RG first interacted with the participants when scheduling and conducting the focus group. Interviews were semistructured using interview guides (available in Multimedia Appendix 1) tailored to each group’s intervention level (eg, individuals randomized to level 1 were asked about the website only, etc). Participants were prompted to answer hypothetically if they did not use or recall certain aspects of the program during the study period. Focus groups were recorded and transcribed. Quotations were edited for clarity, and field notes were made after focus groups. Focus groups took place between May and June 2019; the median time from the end of the study to the focus group was 7 months (Table 1). Men received a US $25 gift card for participating in the focus group.

**Table 1:** Self-reported characteristics of 20 men with prostate cancer who participated in a 12-week remotely delivered lifestyle intervention and volunteered for a postintervention focus group.

Characteristics				Level 1 (n=5)	Level 2 (n=4)	Level 3 (n=5)	Level 4 (n=6)	All levels (N=20)
Age at study enrollment (years), median (IQR)				71 (71-74)	73 (69-76)	68 (68-75)	63 (56-70)	70 (66-74)
**Ethnicity, n (%)**								
	White			5 (100)	3 (75)	5 (100)	6 (100)	19 (95)
	Other			0 (0)	1 (25)	0 (0)	0 (0)	1 (5)
Months from intervention end date to focus groups, median (IQR)				7 (7-8)	10 (7-14)	7 (6-12)	6 (6-12)	7 (6-12)
Years from diagnosis to intervention start date^a^, median (IQR)				6 (3-8)	4 (1-24)	7 (3-8)	3 (1-4)	4 (1-8)
BMI (at diagnosis), median (IQR)				28 (22-30)	28 (26-29)	23.1 (22-27)	26 (24-32)	27 (23-29)
**Stage at diagnosis, n (%)**								
	T1			1 (20)	1 (25)	1 (20)	1 (17)	4 (20)
	T2			3 (60)	1 (25)	4 (80)	2 (33)	10 (50)
	T3 or T4			1 (20)	2 (50)	0 (0)	2 (33)	5 (25)
	Unknown			0 (0)	0 (0)	0 (0)	1 (17)	1 (5)
**Gleason score at diagnosis, n (%)**								
	<7			1 (20)	1 (25)	1 (20)	2 (33)	5 (25)
	7			2 (40)	1 (25)	2 (40)	2 (33)	7 (35)
	>7			1 (20)	2 (50)	2 (40)	2 (33)	7 (35)
	Unknown			1 (20)	0 (0)	0 (0)	0 (0)	1 (5)
**Prostate-specific antigen level (ng/mL), median (IQR)**								
	At diagnosis			4.5 (4.0-10.0)	5.0 (3.7-9.0)	4.9 (4.0-6.1)	11.6 (6.0-14.0)	6.0 (4.0-11.6)
	Most recent			0.4 (0.1-1.0)	0.0 (0.0-0.2)	0.1 (0.1-1.0)	0.1 (0.0-0.2)	0.1 (0.0-0.7)
**Treatment type, n (%)**								
	Radical prostatectomy			2 (40)	3 (75)	2 (40)	3 (50)	10 (50)
	Radiation			2 (40)	3 (75)	2 (40)	4 (67)	11 (55)
	**Medical management**			1 (20)	1 (25)	0 (0)	1 (17)	3 (15)
		**Androgen deprivation therapy**		1 (25)	1 (25)	0 (0)	2 (34)	3 (15)
			Abiraterone acetate	0 (0)	0 (0)	0 (0)	1 (17)	N/A^b^
			Enzalutamide	1 (25)	0 (0)	0 (0)	0 (0)	N/A
			Leuprolide acetate	0 (0)	1 (25)	0 (0)	1 (17)	N/A
		Immunotherapy (Sipuleucel-T)		0 (0)	0 (0)	0 (0)	0 (0)	0 (0)
		Chemotherapy		0 (0)	0 (0)	0 (0)	0 (0)	0 (0)
	Active surveillance			0 (0)	0 (0)	1 (20)	0 (0)	0 (0)
	Other (ie, Radium 223)			0 (0)	0 (0)	0 (0)	0 (0)	0 (0)
**Comorbidities**								
	Total number, median (IQR)			4 (3-7)	4 (2-6)	3 (3-3)	2.5 (1-4)	3 (2-5)
	**Any, n (%)**			4 (80)	3 (75)	5 (100)	6 (100)	18 (90)
		Heart related^c^		4 (80)	1 (25)	4 (80)	3 (50)	12 (60)
		Lung related^d^		0 (0)	0 (0)	0 (0)	2 (33)	2 (10)
		Other^e^		4 (80)	3 (75)	4 (80)	4 (67)	15 (75)

^a^Year of diagnosis only reported for 4 men in level 1, 3 men in level 2, 3 men in level 3, and 5 men in level 4.

^b^N/A: not applicable.

^c^Heart-related comorbidities include hypertension, angina, congestive heart failure, heart attack, irregularity, stroke, peripheral vascular disease, and deep vein thrombosis.

^d^Lung-related comorbidities include chronic obstructive lung disease, acute respiratory distress syndrome, emphysema, and asthma.

^e^Other comorbidities include diabetes, neuropathy, hernia, hearing impairment, arthritis, osteoporosis, and back issues.

### Grounded Theory Analyses

We used a grounded theory approach [39,40]. Coding was completed manually by one investigator (EW) and reviewed with 4 other investigators (SK, JB, RG, and EV); axial codes were managed in Microsoft Excel.

**Figure 3:**
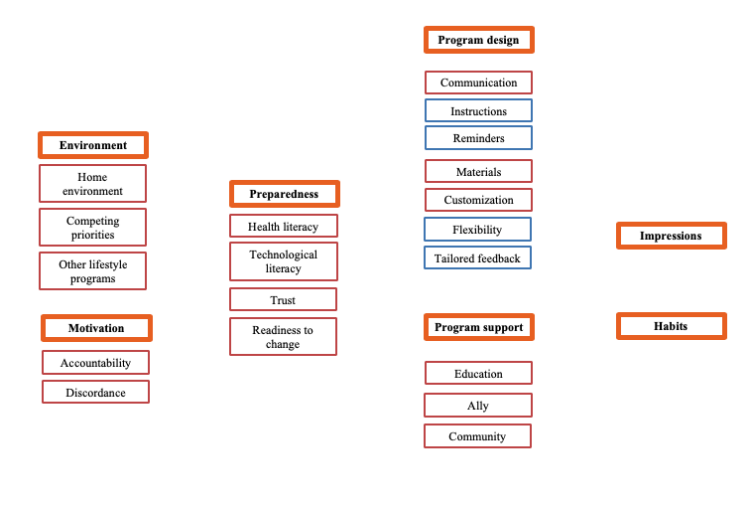
Codes developed using grounded theory analysis: open codes (blue), open codes elevated to axial codes (red), codes elevated to selective codes or categories (orange).

We conducted open, axial, and selective coding (Figure 3). Open, line-by-line coding generated data-driven codes that were refined into 15 axial codes. Ultimately, through embodied categorization [41] and constant comparative methods (to address the multiple levels) [42], we consolidated the data under 7 selective codes (categories). From these categories, a grounded theory surrounding prostate cancer survivors’ use of web-based lifestyle interventions emerged. The codes and their relationships to one another were intermittently discussed and finalized among EW, RG, JB, EV, and SK.

## Results

In total, 10% (20/200) men (of pilot study participants) with prostate cancer participated in the focus groups; 5, 4, 5, and 6 men in levels 1, 2, 3, and 4, respectively. The characteristics of the focus group participants are presented in Table 1. The participants were predominantly White and aged >70 years. The median time from diagnosis to intervention start date was 4 years, and the median time from pilot study intervention end to focus groups was 7.3 months. The median BMI of the focus group participants was 26.6 kg/m^2^ (IQR 22.7-29.3). Various prostate cancer grades, stages, and treatments were represented among the participants. The majority of participants reported multiple comorbidities; only 10% (2/20) men reported no comorbidities.

We identified 5 categories influencing intervention engagement: (1) environment (home environment, competing priorities, and other lifestyle programs), (2) motivation (accountability and discordance), (3) preparedness (technology literacy, health literacy, trust, and readiness to change), (4) program design (communication, materials, and customization), and (5) program support (education, ally, and community; Figure 3). We also identified the long-term effects of the interventions (impressions and habits). Each code represents an actionable component contributing to the overall intuitiveness and seamlessness of this web intervention, as demonstrated by participant quotes below.

### Environment

Participants discussed the environmental factors influencing their participation and impressions of the program.

#### Home Environment

Participants’ family members and geographic locations played roles in their perceptions and usage of the web-based program:

I’ve been thinking about this a little bit and the food groups and what’s best and what’s less good for us is helpful and it’s interesting to me, however, real issues are almost barriers to changing diet. Those can be from things that we don’t have much control over at all like when we’re travelling, restaurants typically don’t have the best food, I will say. And sometimes at home, especially for us guys, I think there’s an element of gender issue here but in my situation I’m the eater and my wife is the cooker. She needs to be part of this somehow.

Participant 2, level 1, aged 74 years, 4 years since diagnosis

I looked at [the website] several times and gave me some ideas and stuff. I had joined a fitness club at one time, so it kind of brought back up some of those exercises to my program here. So, we’re not close to a gym here. Where I live it’s a small community. So, we just do our walking and biking on our own.

Participant 2, level 2, aged 66 years, 1 year since diagnosis

#### Competing Priorities

Participants had multiple other commitments often related to their health care. These limited the amount of time and engagement with the web-based program:

For whatever reason, I don’t know, I didn’t engage with the program. I live a fairly busy life. I’m the president of our local running club and involved in sailing and so many active things that I rarely, other than seeing my medical providers, of which there are so many at this point, I just didn’t engage and I don’t know why, I didn’t.

Participant 2, level 1, aged 74 years, 4 years since diagnosis

I have to admit that I’m a little confused about how this study, the exercise and diet study relates to the surveys that I receive periodically from your group. But part of my confusion rests with the fact that I’m probably involved in three or four different studies.

Participant 3, level 2, aged 79 years, 24 years since diagnosis

When people are going...through radiation, going through post radiation, you know, with being tired, whatever, you tend to just kind of space on things. Particularly if you’re being jacked up with hormone therapy too. You get kind of fuzzy and you don’t sit there and pay as much attention as you might.

Participant 3, level 4, aged 70 years, 3 years since diagnosis

#### Other Lifestyle Programs

Participants frequently reflected on components of the web-based program with reference to previous experiences with weight loss programs and other wearable technology. This influenced the attitudes they carried into the program:

...there’s several programs you can get on your phone and computer who do the same thing and I’ve actually tried one or two of them in the past and kept up with it for maybe two days and that’s it.

Participant 5, level 1, aged 80 years, 8 years since diagnosis

I’ve consulted a nutritionist in the past and probably could use that.

Participant 2, level 1, aged 74 years, 4 years since diagnosis

I think a cooperative with some of those food services might be something to look at. Obviously, not everybody can do that. But that was a thought.

Participant 2, level 4, aged 53 years, 1 year since diagnosis

### Motivation

Participants discussed factors influencing their motivation to participate in the program.

#### Accountability

Participants described or alluded to a sense of accountability:

We lie to ourselves about how we’re doing. But heart rate and other indicators are hard to fool so I’ve actually discovered that I have some other issues through my [own heart rate] monitor and that’s been good.

Participant 2, level 1, aged 74 years, 4 years since diagnosis

Probably a shortcoming on my part. I didn't explore the website nearly as much as I probably should have.

Participant 3, level 3, aged 65 years, years since diagnosis not reported

I can’t remember. But, again, I was also sometimes forgetting. And so there were gaps in the data, and I felt really bad about that. You know? Because I hadn’t realized that I should have connected the day before or something.

Participant 2, level 4, aged 53 years, 1 year since diagnosis

#### Discordance

Participants shared their discordant experiences within the health care system—the web-based intervention occurred amid the background of the confusion that these previous experiences had created:

...urologists...I mentioned sugar to him. He said, no, sugar’s not going to make any difference...he says the only thing that has proven to be of any help is cooked tomatoes. And I mentioned this to a couple of nurses, three different nurses and essentially one nurse...said, doctors don’t know anything about diet... It’d be nice if urologists would somehow send people to someplace like your website.

Participant 1, level 2, aged 73 years, years since diagnosis not reported

You folks are in universities whereas we’re mixing what we get from our doctors as providers with what you folks are doing to study...maybe you could feel free to comment on what the purpose of all this is...Are the people on your staff the ones who would stay with this program for years?

Participant 1, level 3, aged 75 years, 7 years since diagnosis

### Preparedness

In addition to environment and motivation, participants’ unique skill sets and backgrounds influenced their ability to engage with the program.

#### Health Literacy

Participants demonstrated varying levels of health literacy (ability to communicate an understanding about prostate cancer and/or the purpose of the study), which affected their interest and engagement with the program:

When it became evident that prostate cells had escaped prior to surgery and were floating around in my bloodstream somewhere. I guess I never felt that sitting around in a group thing was going to do anything to change that. It was a medical science issue, not a communication issue.

Participant 3, level 2, aged 79 years, 24 years since diagnosis

My primary interest was the diet…I was intrigued to learn so many different ways that diet impacts survivability when you’re diagnosed with cancer. So I really just felt it was important, and that’s when I kind of delved in.

Participant 6, level 4, aged 56 years old, 1 year since diagnosis

#### Tech Literacy

Using a web-based intervention requires some baseline comfort using technology—the participants greatly varied in their preferences, which affected their engagement with the program:

You know I think probably a very natural tendency for all of us, regardless of whether it’s prostate cancer or some other life-threatening disease, we tend to hit the internet, if you will, and look for information. I certainly did that in the beginning.

Participant 1, level 1, aged 65 years, years since diagnosis unknown

Well much to my kids and grandkids consternation, I don’t read text messages.

Participant 4, level 3, aged 84 years old, 8 years since diagnosis

#### Trust

Participants discussed how their trust has been eroded by past experiences with health care:

One of the frustrations that I have of moving around a bit in the country and having to reestablish relationships is always a challenge because quite frankly, the quality of many of the people I’ve had to work with, physicians and all this, sometimes is not very high. And you feel valuable when you’ve found a resource that you can trust, and then to have those people go away is a problem.

Participant 1, level 3, aged 75 years, 7 years since diagnosis

#### Readiness for Change

Participants commented on their readiness for behavioral change and experiences shaping this factor:

I guess I’m addicted. I’m always working towards some goal.

Participant 2, level 1, aged 74 years, 4 years since diagnosis

...I didn’t change much but just this awareness that things need to change. Your diet, and you move around a whole lot more.

Participant 4, level 1, aged 71 years, 1 year since diagnosis

...like most exercise programs, extremely difficult to get the discipline built. And I do recognize that I probably should be doing them, particularly the balance exercises and strength exercises. My diet’s probably not going to change much. I’m reminded of a friend’s father at 95 coming home from the hospital for a heart attack, stopped at a restaurant, and ordered french fries and onion rings. And his son said, dad, you shouldn’t eat like that. He says at 95, he says, what’s it going to do? Kill me? So the tendency with diet I think is to say, yeah I know I shouldn’t...I had to cut back on some of this stuff, but it doesn’t appear to be hurting my health. And maybe that’s a message that somehow you need to deliver more strongly.

Participant 3, level 2, aged 79 years, 24 years since diagnosis

I wouldn’t need [informational text messages] like that because, like I said, I’m doing something on my own already and I’m pretty satisfied with it. But that’s just my feeling about it.

Participant 3, level 3, aged 65 years, years since diagnosis not reported

### Program Design

Participants reflected on the various components of the program and suggested improvements.

#### Communication

Comments about how participants hoped communications would be used and how they might be improved:

As I’m looking at this, I’m a little embarrassed to say I didn’t find this on the website. Maybe one of the messages would have been really helpful to remind me to look here.

Participant 2, level 3, aged 68 years, 3 years since diagnosis

I think it would have been nice to have some kind of a general email once in a while every few weeks or more often, just about this whole thing. You know, kind of reminding us what’s available to us and maybe asking for feedback even then, as human to human.

Participant 1, level 3, aged 75 years, 7 years since diagnosis

So I guess the question I have is when you say “coach,” I’m not clear. Because is the coach acting as the expert, in terms of information? Or are they acting in terms of holding us accountable and giving us that position. So I’m not really...I guess that’s the question, are they there to be the expert role or are they there to be the coach?

Participant 6, level 4, aged 56 years, 1 year since diagnosis

When receiving the text messages, they came from different numbers...if I was to keep them, I had to kind of keep this whole catalog of texts from different phone numbers. So, if it’s possible to standardize the messaging from one sourced number, it would be easier to just have a ready reference for all the information that was provided...I think they’re worthy of keeping.

Participant 6, level 4, aged 56 years, 1 year since diagnosis

#### Materials

Participants discussed the program materials (recipes, in print vs on the web, and wearable technology):

...again, if I think about different diet programs, they give you the ability to find creative substitutes and creative, not just recipes but be able to say I’m looking for some creative alternatives for when I’m lunch on the go or something like that.

Participant 1, level 1, aged 65 years, years since diagnosis unknown

I’ve mastered the ability to print almost anything displayed on a website. So, I don't need to have a mailing. If it's available on the website and I wanted it in print, I can make that happen.

Participant 3, level 2, aged 79 years, 24 years since diagnosis

I found [the Fitbit] very unhelpful. Number one, I don't know how to read it, and it was hard to put on with one hand.

Participant 4, level 3, aged 84 years, 8 years since diagnosis

Yeah I think the Fitbit is a little behind. I think as I’d mentioned, the Oura is probably a better route to take. It’s just on your fingers. You don’t have to worry about it...And to the activities that it doesn’t auto-recognize or automatically sync on, you do have to go in there, as you would with any other wearable tech, you do have to go in and kind of manipulate that and add that to it.

Participant 6, level 4, aged 56 years, 1 year since diagnosis

#### Customization (Flexibility)

Participants of all levels commented on their desire for increased customization and flexibility—many participants mentioned that their engagement in various aspects of the program would have changed if messaging delivery or content was customized:

If we were talking about things that were targeted based on my activity on the site or my filters or my preferences I might say more often but if it’s just more general type information, weekly [text message reminders] would probably be good.

Participant 1, level 1, aged 65 years, years since diagnosis unknown

...as I recall, the prescription was developed based on a questionnaire that I had submitted to you prior to the beginning or at the beginning of the study. So it at least purported to be specific recommendations to the lifestyle and concerns that I as an individual had in that sense. If that’s correct, then it might be helpful to have the opportunity to periodically develop a new prescription to answer the same questionnaire submitted...it would be helpful maybe every six months or so to give participants the opportunity to complete the questionnaire again with updated information and develop a new prescription.

Participant 3, level 2, aged 79 years, 24 years since diagnosis

I’ve had a couple bouts with heart failure, so right now I’m on a salt-free diet and it would be helpful to me to be a little more specific as to what I can eat and what I can’t eat regarding that particular restriction.

Participant 4, level 3, aged 84 years, 8 years since diagnosis

But I think that his idea of having more flexibility is a good one. Being able to tailor it to your particular lifestyle would be beneficial as well.

Participant 3, level 3, aged 65 years, years since diagnosis not reported

Yeah, and I think [the text messages] were pretty good, even as generic as they were, just to be a reminder and motivator.

Participant 2, level 4, 53 years old, 1 year since diagnosis

#### Customization (Tailored Feedback)

The participants commented on the benefits of tailored immediate feedback for meeting their lifestyle goals:

I like [the surveys] because the feedback was immediate and I could put it in and just right away I knew where I was, where I stood as far as doing good or not doing good and I liked that process.

Participant 2, level 2, aged 66 years, 1 year since diagnosis

I thought it was useful. Like I said, it’s kind of a dialogue. It tells you whether you’re doing what you should be doing or not, to get the feedback, immediate feedback.

Participant 5, level 4, aged 78 years, 16 years since diagnosis

I think if [the website] worked in tandem with the coaching process, maybe there would be more visibility on. And so, in terms of that being helpful, yes, I think either you go in, you look, you work with your coach, you see there’s a dip...if you convert that sole tool from an extrinsic motivator to more of an intrinsic motivator when you're working with somebody to help you see the benefit of moving through your exercise regime and getting stronger. Right? And so, I think it would work well if you paired it with the coaching process.

Participant 6, level 4, aged 56 years, 1 year since diagnosis

### Program Support

Participants communicated their expectations of various types of support from the web-based program.

#### Education

Participants from all levels provided suggestions on how to improve the educational component of the intervention:

What I did like about this particular site and participation in this was I felt like I was getting consistent information across diet, diagnosis, symptoms, side effects, and so forth.

Participant 1, level 1, aged 65 years, years since diagnosis unknown

I guess you could put more links in to connect us to information. I mean there’s stuff I have to go searching for on the internet anyway, but you put that information in the stuff that you send to us it might save us a little time...Anything about the disease and its cure. I mean the amount of information available on the internet about prostate, it’s almost like drinking out of a fire hydrant. If there’s anything special that...you want to make people aware of, that would be good.

Participant 5, level 1, aged 80 years, 8 years since diagnosis

Anyway, I’d like to see if there was someplace, if I had question, the food and the exercise, if somewhere I could easily go to another website or get these studies that prove [inaudible] is good to prevent cancer. You’re just telling us...I’m following it. You’re just telling us don’t do this, do this, this, this, this. Without any resources to back it. I’m not seeing the...studies or how extensive [a] study was.

Participant 1, level 2, aged 73 years, years since diagnosis not reported

Being a non-cooking person which I’m trying to change...I wasn’t sure what a cruciferous vegetable was when we started, so just having a list of cruciferous vegetables...So I was just looking for additional resources. In some cases, some ideas in terms of cooking or putting food together, some of those I shared with my wife, some of them were just looked at. You know, when you say, “Eat more fish.” It’s not really about eating more fish, it was about eating more salmon and related fish in terms of oils. So that type of stuff helped.

Participant 2, level 3, aged 68 years, 3 years since diagnosis

So, the internet is full of information. Some of it really helpful, some of it really pretty horrid. As part of the resource would be some direction in terms of, “Here’s some places you can go to get some really good information about this that might be outside OHSU [Oregon Health and Sciences University]...”

Participant 2, level 3, aged 68 years, 3 years since diagnosis

Well as I’m looking at it now, it seems to mostly like recipes and things like that. I would be much more interested in technical information about cancer or exercise or something of that sort.

Participant 5, level 4, aged 78 years, 16 years since diagnosis

I was actually drawn to the diet piece. There was actually some very helpful and not helpful bits of information, like the gentleman that raised the topic of tomatoes. I went down that path and incorporated tomatoes, cooked tomatoes, some ripened tomatoes, all the different types of salsa. Things that really made the meal at some points. And so I thought that was really helpful.

Participant 6, level 4, aged 56 years, 1 year since diagnosis

#### Ally

Participants wanted someone who genuinely cared about their progress available to answer questions and provide support:

Just a couple thoughts on coaches. I think it’s definitely helpful to have more of a personal interaction. You know, with the coach giving reminders, as opposed to having an email message kind of a reminder coming from a program. You know, if you have that more personal...Someone that’s interested in what you’re accomplishing, I think that’s a better motivator.

Participant 1, level 4, aged 56 years, years since diagnosis not reported

The promise of a coach is somebody who can celebrate with you when you’ve reached your goals...and can also listen to you when you’re struggling and be empathetic.

Participant 2, level 4, aged 53 years, 1 year since diagnosis

Ideally, the coach should provide both functions. He should have deep expertise and be a motivator, just like a football coach.

Participant 5, level 4, aged 78 years, 16 years since diagnosis

#### Community

An overwhelming majority of participants appreciated having others with similar experiences to relate with:

...when I was first diagnosed with prostate cancer, I went to a local support group of meetings and it was really terrific. The ability to interchange information, there’s no substitute for it as far as I'm concerned and if there was a way you could enable that I’d be all for it.

Participant 5, level 1, aged 80 years, 8 years since diagnosis

I think [Community of Wellness] is perfect because there’s so many different...some people are doing active surveillance, some people are doing radiation, some people are just...there’s so many different things but does anybody have the real answer of what worked for them or what is working for you, that’s hard to do.

Participant 4, level 1, aged 71 years, 1 year since diagnosis

[Community of Wellness] is just a way to interact with people who are going through the same thing, and sometimes get support and sometimes receive it through that kind of community.

Participant 1, level 3, aged 75 years, 7 years since diagnosis

One of the things I really liked about [Community of Wellness] was that I felt like I was part of a community. Not only was it in the name, but it was nice to feel like there was some help more than just going to the doctor. So that was very valuable to me. Obviously, there were a lot of benefits that I got from it. You know, maybe they’ll come out during the discussion. I just thought it was really good being a part of something that at least acknowledged, “Hey, we’re alive. We have cancer, but somehow, we’re getting through it,” and that sort of thing was emotionally quite beneficial.

Participant 1, level 3, aged 75 years, 7 years since diagnosis

### Impressions and Habits

Ideally, lifestyle interventions help participants develop lifelong habits. In this quotation, one participant offers his thoughts on the long-term impacts of this intervention. This quotation and others reflect the participants’ impressions of the program; these impressions add to their collective experiences with technology and health:

six months later...I have really changed. [The program] kind of kicked it off. But if I look back from where I am right now, and what I’m eating now, and how I’m eating, it’s dramatically different than how I was before I entered the program, and even when I finished the program because I continued on that trajectory, and been able to really, to do that. So, I think as far as coaching goes, everybody’s different. And I’m not sure that the program’s long enough to be able to really drive the kind of...You know, to be able to see the sustained change, or even get to the sustained change, maybe.

Participant 2, level 4, aged 53 years, 1 year since diagnosis

### Intuitive Interventions

Each code generated in this study represents a unique mechanism for designing a more intuitive, web-based lifestyle intervention for prostate cancer survivors. By addressing the environment, we may transform factors that already exist in participants’ lives as obstacles to reinforcing factors for improved engagement with the web-based program. By addressing participants’ motivation, we may improve our ability to tailor web content and web-based communications. Understanding participants’ preconceived attitudes based on past encounters with the health care system will allow us to actively address concerns and improve program adherence. We may influence preparedness when we assess and consider each participant’s unique level of health and technological literacy, readiness to change, and trust and bolster these whenever possible through program content. Program design and program support are the most easily affected; we can increase intuitiveness through tailored communication, materials, and feedback, providing quality educational content, serving as allies, and generating community.

Noting the ways in which certain codes presented in the different intervention levels helped contextualize feedback. For example, participants in level 1, who received only web access to educational content, requested more communication, whereas participants in levels 2 to 4, who received increasing levels of behavioral support, provided details on ways in which the multiple forms of communication they received might be tailored. Participants in levels 3 and 4 received more types of behavior support and were also more likely to request more instructions or reminders orienting them to the program, as their interventions had more components. Conversely, some codes were commonly expressed across groups, such as competing priorities, readiness for change, flexibility, education, and community.

**Figure 4:**
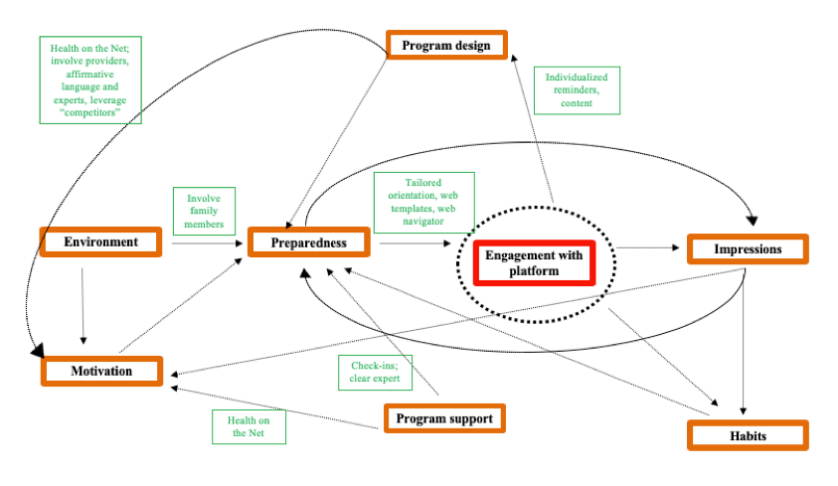
Grounded theory-based approaches to increasing prostate cancer survivors’ engagement with web-based Community of Wellness lifestyle intervention: relationships among barriers and motivators related to engagement with web-based behavioral support, with potential solutions (green, ie, Health on the Net).

The relationships among these codes (Figure 4 [41]) represent iterative, actionable pathways by which designers may increase program intuitiveness for prostate cancer survivors engaging in web-based interventions often via multiple mechanisms at once. For example, we might influence motivation (accountability and discordance) by improving program design in the following ways: (1) using Health on the Net [43] transparency and quality principles (quality, confidentiality, neutrality, transparency, community, and visibility) for certification, (2) communicating with clinical providers about participants’ involvement in the program, (3) remaining sensitive to participants’ guilt with failures to modify behaviors, and (4) leveraging participants’ familiarity with existing lifestyle programs to optimize engagement. These and other grounded theory–based solutions (Table 2) may result in a more accessible and integrated intervention for prostate cancer survivors.

**Table 2:** Participant-inspired recommendations to improve intuitiveness and engagement with remotely delivered behavioral interventions for men with prostate cancer.

INSERT	Issue	Solution	Recommendations for improvements
Environment	Home environment Competing priorities Other lifestyle programs	Anticipate and leverage potential sources of friction preventing participation Involve providers Involve family members	Send letters framed toward stakeholders’ unique role in the patient’s program involvement Leverage existing programs (eg, partner with meal delivery services and/or gyms with discounts for patients with cancer)
Motivation	Accountability Discordance	Provide longitudinal support Minimize stigma	Provide quality feedback or monitoring Continue to use judgment-free language
Preparedness	Health literacy Technological literacy Trust Readiness for change	Assess patient comfort level with technology Assess health literacy Assess readiness to change	Use tailored web templates based on technological and health literacy Incorporate customizable web interfaces Customize orientation to program Incorporate website navigator Use motivational interviewing techniques to assess baseline readiness and subsequent progression
Program design	Communication (instructions and reminders) Materials Customization (flexibility and tailored feedback)	Maximize relevant information Minimize extra information	Construct and use individual profiles per baseline, performance, and other time commitments Add individualized reminder content and frequency Create various versions of the site to match health and technological literacy of the user
Program support	Education Ally Community	Improve transparency Increase ally availability	Add Health on the Net certification Emphasize “coach’s” role as expert and support person Allow for updates to profile Add ability to filter resources

## Discussion

### Implications

Men with prostate cancer find themselves in an era of seemingly limitless access to medical information via the web. Technological advances impact their daily lives, and as technology and health care delivery are increasingly intertwined, their ability to maintain health may inevitably be influenced by their willingness to engage with technological interfaces [44,45]. We learned that prostate cancer survivors within this study were sensitive to discrepancies related to clinical evidence and practice. They developed heuristics for navigating copious information, they described an interest in transparent sources, and they voiced a desire for continuity and ongoing care. They discussed the emotional impact of their participation within the health care system; these cumulative experiences (including newer experiences with technology-based care) underlie all experiences with health-promoting interventions.

### Qualitative Methodology

We used the grounded theory methodology because no comprehensive theory of web interventions for behavior change in prostate cancer survivors has been developed before. This methodologic approach is a strength because data-driven open coding is most equipped to interrogate the inherent assumptions held by study participants and researchers alike [40]. Another strength of the study was the interpretation of data across groups receiving progressive levels of lifestyle interventions.

### Comparison With Prior Work

An intuitive, web-based interface is not a novel concept. In 1993, Nielsen [46] coined the term *usability engineering*, where the usability of a system is defined by (1) learnability, (2) efficiency, (3) memorability, (4) low error rate, and (5) satisfaction. Usability heavily overlaps with intuitiveness, although we believe intuitiveness emphasizes tailoring and program responsiveness, shifting the burden of anticipation on program designers rather than program users. The interest in temporal and user tailoring beyond usability is also illustrated by the growing literature on *just in time* adaptive interventions, which are designed to adapt according to changes in an individual’s contexts over time. These interventions provide the most appropriate and timely support to their users (usually enabled by mobile and sensing technologies); their applications in health promotion are of particular interest [47].

Our findings suggest that intuitiveness will likely depend on both the context and the intended user. This qualitative study elucidates some of the key areas that can be optimized for intuitive use of an internet-based lifestyle intervention among well-educated, White prostate cancer survivors. Although we used a grounded theory approach and generated data-driven codes, many of the resulting codes and their relationships to one another (Figure 4) are corroborated by existing theories in public health, as described below.

The environment code (applied in instances where participants mention environmental factors impacting their program engagement) is corroborated by the idea of a multilevel intervention based on the social ecological model. The social ecological model by Bronfenbrenner and Morris suggests that the individual is enveloped and influenced by interpersonal, organizational, community, and public policy networks [48]. *Readiness to change* is supported by the transtheoretical model stages of change (with the stages of precontemplation, contemplation, preparation, action, maintenance, and termination) [49]. The idea that self-efficacy and agency influence how accountability is achieved (social cognitive theory) is highly consistent with motivation (accountability and discordance) [50]. Finally, the health belief model [51], which differentiates between behaviors in health and illness, is especially interesting when applied to lifestyle interventions in prostate cancer survivors. Prostate cancer survivors are in a unique position of having a chronic illness but also being in a position to engage in preventative health behaviors to deter recurrence or disease progression. The various components of the health belief model (perceived benefits vs perceived threat, self-efficacy, and cues to action) are impacted by the large majority of codes in our grounded theory model.

### Limitations

Limitations of the study include the small subgroup sample size and lack of a theoretical sampling process parallel to the analyses. Overall, 10% (20/200) of eligible men were both interested and available to participate in the focus groups at the scheduled times. Although the smaller sample size is acceptable as our objectives were to explore themes using a grounded theory approach, this introduces a possible selection bias. In addition, not all participants fully participated in the web intervention as indicated, and the focus groups took place a median of 7 months after the interventions. Some men participated in multiple TrueNTH programs or were involved in other clinical trials. Although the longer follow-up period and competing priorities contributed valuable, realistic insight into the participants’ lasting impressions and their habit formation, participants may not have recalled all the details of the intervention. In addition, although this was a multi-institutional study, the participants’ experiences may primarily reflect viewpoints of educated, White men in the West and Mountain regions of the United States, where there may be disproportionately greater exposure to technology and overall better physical activity rates [52]. The lack of theoretical sampling and smaller subgroup sample size limits our ability to confidently comment on data saturation. In response to these limitations, we had a low threshold to include open codes in grounded theory, even if they were introduced by just 1 or 2 participants (ie, preparedness: trust, impressions, and habits); data-driven codes were also more likely to be elevated to axial or selective code status if the concepts they represented were supported by previous well-supported theories in public health. This qualitative study does not provide insight into which level of intervention performed best for this group of end users; however, it does provide researchers with important insights into the challenges of creating web-based approaches to support survivorship care that is both high tech and accessible. Further quantitative studies are needed to confirm the validity and directionality of these associations. Further work is needed to explore how our proposed theory applies to men with different sociodemographic characteristics.

### Conclusions

Our study demonstrates that a web-based lifestyle intervention for men with prostate cancer can become intuitive and encourage adherence. These include addressing technological and health literacy, motivation, and environmental factors. In addition, flexible and transparent web design, integration of key stakeholders (ie, providers, family members), and effective coaching may improve the usability and intuitiveness of a web-based intervention to support prostate cancer survivorship. Men with prostate cancer tend to be older, have comorbidities, and balance multiple priorities; this may limit their ability to engage with a web-based lifestyle platform. A web intervention’s potential to affect long-term change will depend on the intuitiveness of its components, allowing integration within an individual’s daily life (eg, clinical support, familial involvement, preparedness for program participation). This grounded theory–based analysis may help guide future web intervention designs for cancer survivors. The convergence of our findings with well-established theories in public health suggests that certain aspects of our theory are broadly applicable to lifestyle intervention design, although this will require further study.

## Appendix

Multimedia Appendix 1 Focus group guides for Community of Wellness.
