# Supplementary material for: Web-Based Lifestyle Interventions for Prostate Cancer Survivors: Qualitative Study
Source: JMIR Cancer. 2020 Nov 10;6(2):e19362. doi: 10.2196/19362 (PMC7685923; doi:10.2196/19362)
Supplement: Multimedia Appendix 1 [file cancer_v6i2e19362_app1.pdf]

Focus Group Guide for Community of Wellness: Level 1

I. WELCOME AND INTRODUCTION (10 minutes; 10 minutes passed)
Welcome! Thank you all so much for coming. My name is Rebecca Graff and I will be the facilitator of this discussion group. We've invited you here to learn from you. Hearing about your experiences, ideas and opinions about the Community of Wellness study will help us to further develop materials and programs that will be useful to others who have gone through the same experiences as you, so we really appreciate you taking the time to come talk with us.

Before we begin, I'd like to walk through some key points about today's session:

A. Opinions:
The questions we will ask will be pretty straightforward. There are no right or wrong answers; we are interested in hearing what you think and what experiences you have had. All comments--both positive and negative--are needed. Please feel free to disagree with one another, but please don't criticize other people's opinions. Please also speak one at a time so that we can listen to your opinions.

B. Confidentiality:
- With your permission, we would like to record the conversation so that we can accurately capture all of your ideas.
- Taking part in this group is totally voluntary. You can leave at any time or not answer any question for any reason.
- All the information you provide will be kept confidential and will only be used by the people directly involved with this project. Your real names will not be used in any reports or publications about this research.
- If you need to refer to yourself in the third person, please only use your initials.
- Do not tell anyone outside the group what any particular person said in the group.

C. Housekeeping:
- Our discussion will last between 60 and 90 minutes.
- I really want to hear from each of you, so I hope that you will all feel comfortable voicing your opinions. Try to speak one at a time and be advised that I may call on particular people to ensure that we're hearing from everyone.
- I have some colleagues who will be listening in today. They're also interested in hearing your thoughts and feedback, but you won't be interacting with them directly.
- You can message the group using the "Chat" function or you can message an individual. To make sure that you know how to use the function, go ahead and send a message to

the group. Now send a message to me only. If you need to take a break or want to tell me anything confidentially, go ahead and send a direct chat to me.

- Can everyone see my screen?
- Are there any questions for me before we begin?

D. Introductions:

- Let's have you very briefly introduce yourselves and tell us where you're from. Please use your initials rather than your name. *[Suggest someone to begin.]*

II. Impressions of Study (10 minute; 20 minutes passed)

1. To begin, I'd like to hear about your experience with the Community of Wellness study. Overall, what were your impressions?
2. To what extent, if any, did you change your exercise or dietary habits during the study?
    a. How did you go about making the changes?
    b. What were the easiest changes for you to make? The hardest?
    c. What, if anything, made it difficult to make changes?
        i. Probe: Physical limitations, social support, cost, time, lack of knowledge of proper diet/exercise
    d. Since finishing the study do you feel that you have been able to sustain these changes? Why or why not? What would help you sustain healthy eating and exercise habits?
3. Which components of the study, if any, were the most effective in helping you to make diet or exercise changes?
    a. Did you feel that the study was missing anything that would have been especially helpful or would have motivated you more to make diet or exercise changes?
4. Did participation in the study ever feel time-consuming or burdensome? If yes, why?
5. What do you feel was the most important thing that you learned from participating in the study?

III. Overall Experience with Website (10 minutes; 30 minutes passed)

Now I'd like to discuss the website that was part of the study.

6. Did you access the website during the study? How often?
7. If you went to the website, did you access it on a computer, tablet or phone? What would your preferred platform be for future versions of the website?
8. What triggered you to use the website? (I.e., when did you use it?)
9. What did you like most about the website?
10. What did you like least or what was most difficult to use on the website?
    a. What is one thing you think we MUST change or do to improve this site?
11. Did you show the website to others or give a family member your login information?
    a. Do you think it would be useful to give family members logins? Why or why not?
    b. Should the website have a page with content tailored to family members or significant others? What kind of information do you think would be useful on such a page?
12. Is there anything else that you would have liked to have seen on the website?

a. *Probe for: More support info about cancer treatment/management, blog posts, other types of exercise content*

IV. Basic Website Components (20 minutes; 50 minutes passed)
At this point, I'd like to bring ask you about some specific components of the website as I show them on my screen.

13. Currently we're looking at the dashboard page of the website.
    a. How often, if at all, did you interact with this page?
        i. For those who didn't interact with this page, why not?
    b. What, if anything, you do like about this page?
    c. What, if anything, would you change about this page?
        i. *Probe about alternative formats (e.g., PDF, text messages, interactive app, etc.)*
    d. Is there any additional information that you would like to see on this page?
14. Now let's move on to the Exercise Info page of the website.
    a. How often, if at all, did you interact with this page?
        i. For those who didn't interact with this page, why not?
    b. What, if anything, you do like about this page?
    c. What, if anything, would you change about this page?
        ii. *Probe about alternative formats (e.g., PDF, text messages, interactive app, etc.)*
    d. Is there any additional information that you would like to see on this page?
15. Now we're looking at the Diet Info page of the website.
    a. How often, if at all, did you interact with this page?
        iii. For those who didn't interact with this page, why not?
    b. What, if anything, you do like about this page?
    c. What, if anything, would you change about this page?
        iv. *Probe about alternative formats (e.g., PDF, text messages, interactive app, etc.)*
    d. Is there any additional information that you would like to see on this page?
16. Let's now jump to the Resources page of the website.
    a. How often, if at all, did you interact with this page?
        v. For those who didn't interact with this page, why not?
    b. What, if anything, you do like about this page?
    c. What, if anything, would you change about this page?
        vi. *Probe about alternative formats (e.g., PDF, text messages, interactive app, etc.)*
    d. Is there any additional information that you would like to see on this page?
    e. For those who used the recipes, how did it go? Did you share w/ family member?
        i. Do you typically use recipes? How did our recipes compare?
        ii. *Probe: would it be better to give you one PDF recipe booklet that you could give to a partner or whoever cooked, if not yourself?*
        iii. For those who didn't use the recipes, why not? Generally don't use recipes, or were these recipes not appealing for some reason?

f. What about the shopping guides for each of the foods?
    i. Why/why not? How did it go?
    ii. Are the food decisions and cooking (shopping, choosing the menu, preparing food) shared by a partner? How can we support the shared shopping/cooking responsibilities within the website?

V. Future Directions (15 minutes; 65 minutes passed)

17. How likely or unlikely would you be to use a website feature that allows you to track your diet and exercise?
    a. Which, if any, parts of your diet and exercise would be interested in tracking?
    b. Have you ever previously used a tool to track your diet or exercise? Which one(s)?
    c. Would you prefer to log diet/exercise somewhere else - such as using a paper diary?
18. What do you think about patient websites where you can communicate with other people like you? If we added a feature where you could communicate anonymously with other study members (for example, within a forum on a specific topic), do you think you would use it? Why or why not?
19. [Levels 1-3 Only] If you were offered a 30 min diet or exercise phone counseling session, would you use it? If you could ask questions online to a diet or exercise coach at any point during the study, would you this feature?

VI. Wrap-up (5 minutes; 70 minutes passed)

20. Is there anything else you wanted to say in closing that you haven't yet had the opportunity to say?
21. You all have my email address, so don't hesitate to reach out if you have any additional thoughts that you'd like to share.

Thank you for your time and participation.

Focus Group Guide for Community of Wellness: Level 2

I. WELCOME AND INTRODUCTION (10 minutes; 10 minutes passed)
Welcome! Thank you all so much for coming. My name is Rebecca Graff and I will be the facilitator of this discussion group. We've invited you here to learn from you. Hearing about your experiences, ideas and opinions about the Community of Wellness study will help us to further develop materials and programs that will be useful to others who have gone through the same experiences as you, so we really appreciate you taking the time to come talk with us.

Before we begin, I'd like to walk through some key points about today's session:

A. Opinions:
The questions we will ask will be pretty straightforward. There are no right or wrong answers; we are interested in hearing what you think and what experiences you have had. All comments--both positive and negative--are needed. Please feel free to disagree with one another, but please don't criticize other people's opinions. Please also speak one at a time so that we can listen to your opinions.

B. Confidentiality:
- With your permission, we would like to record the conversation so that we can accurately capture all of your ideas.
- Taking part in this group is totally voluntary. You can leave at any time or not answer any question for any reason.
- All the information you provide will be kept confidential and will only be used by the people directly involved with this project. Your real names will not be used in any reports or publications about this research.
- If you need to refer to yourself in the third person, please only use your initials.
- Do not tell anyone outside the group what any particular person said in the group.

C. Housekeeping:
- Our discussion will last between 60 and 90 minutes.
- I really want to hear from each of you, so I hope that you will all feel comfortable voicing your opinions. Try to speak one at a time and be advised that I may call on particular people to ensure that we're hearing from everyone.
- I have some colleagues who will be listening in today. They're also interested in hearing your thoughts and feedback, but you won't be interacting with them directly.
- You can message the group using the "Chat" function or you can message an individual. To make sure that you know how to use the function, go ahead and send a message to

the group. Now send a message to me only. If you need to take a break or want to tell me anything confidentially, go ahead and send a direct chat to me.

- Can everyone see my screen?
- Are there any questions for me before we begin?

D. Introductions:

- Let's have you very briefly introduce yourselves and tell us where you're from. Please use your initials rather than your name. *[Suggest someone to begin.]*

II. Impressions of Study (10 minute; 20 minutes passed)

1. To begin, I'd like to hear about your experience with the Community of Wellness study. Overall, what were your impressions?
2. To what extent, if any, did you change your exercise or dietary habits during the study?
    a. How did you go about making the changes?
    b. What were the easiest changes for you to make? The hardest?
    c. What, if anything, made it difficult to make changes?
        i. *Probe: Physical limitations, social support, cost, time, lack of knowledge of proper diet/exercise*
    d. Since finishing the study do you feel that you have been able to sustain these changes? Why or why not? What would help you sustain healthy eating and exercise habits?
3. Which components of the study, if any, were the most effective in helping you to make diet or exercise changes?
    a. Did you feel that the study was missing anything that would have been especially helpful or would have motivated you more to make diet or exercise changes?
4. Did participation in the study ever feel time-consuming or burdensome? If yes, why?
5. What do you feel was the most important thing that you learned from participating in the study?

III. Overall Experience with Website (10 minutes; 30 minutes passed)

Now I'd like to discuss the website that was part of the study.

6. Did you access the website during the study? How often?
7. If you went to the website, did you access it on a computer, tablet or phone? What would your preferred platform be for future versions of the website?
8. What triggered you to use the website? (I.e., when did you use it?)
9. What did you like most about the website?
10. What did you like least or what was most difficult to use on the website?
    a. What is one thing you think we MUST change or do to improve this site?
11. Did you show the website to others or give a family member your login information?
    a. Do you think it would be useful to give family members logins? Why or why not?
    b. Should the website have a page with content tailored to family members or significant others? What kind of information do you think would be useful on such a page?
12. Is there anything else that you would have liked to have seen on the website?

    a. *Probe for: More support info about cancer treatment/management, blog posts, other types of exercise content*

IV. Basic Website Components (20 minutes; 50 minutes passed)
At this point, I'd like to bring ask you about some specific components of the website as I show them on my screen.

13. Currently we're looking at the dashboard page of the website.
    a. How often, if at all, did you interact with this page?
        i. For those who didn't interact with this page, why not?
    b. What, if anything, you do like about this page?
    c. What, if anything, would you change about this page?
        i. *Probe about alternative formats (e.g., PDF, text messages, interactive app, etc.)*
    d. Is there any additional information that you would like to see on this page?
14. Now let's move on to the Exercise Info page of the website.
    a. How often, if at all, did you interact with this page?
        i. For those who didn't interact with this page, why not?
    b. What, if anything, you do like about this page?
    c. What, if anything, would you change about this page?
        ii. *Probe about alternative formats (e.g., PDF, text messages, interactive app, etc.)*
    d. Is there any additional information that you would like to see on this page?
15. Now we're looking at the Diet Info page of the website.
    a. How often, if at all, did you interact with this page?
        iii. For those who didn't interact with this page, why not?
    b. What, if anything, you do like about this page?
    c. What, if anything, would you change about this page?
        iv. *Probe about alternative formats (e.g., PDF, text messages, interactive app, etc.)*
    d. Is there any additional information that you would like to see on this page?
16. Let's now jump to the Resources page of the website.
    a. How often, if at all, did you interact with this page?
        v. For those who didn't interact with this page, why not?
    b. What, if anything, you do like about this page?
    c. What, if anything, would you change about this page?
        vi. *Probe about alternative formats (e.g., PDF, text messages, interactive app, etc.)*
    d. Is there any additional information that you would like to see on this page?
    e. For those who used the recipes, how did it go? Did you share w/ family member?
        i. Do you typically use recipes? How did our recipes compare?
        ii. *Probe: would it be better to give you one PDF recipe booklet that you could give to a partner or whoever cooked, if not yourself?*
        iii. For those who didn't use the recipes, why not? Generally don't use recipes, or were these recipes not appealing for some reason?

f. What about the <u>shopping guides</u> for each of the foods?
    i. Why/why not? How did it go?
    ii. Are the food decisions and cooking (shopping, choosing the menu, preparing food) shared by a partner? How can we support the shared shopping/cooking responsibilities within the website?

V. [Levels 2-4 Only] Tailored Recommendations (25 minutes; 75 minutes passed)

As part of the study, you were provided with personalized exercise and diet recommendations.

17. How did you feel about the exercise recommendations?
    a. Did you follow the recommended exercise program? What was challenging about doing the exercise program?
    b. Did you read and use the safety information?
    c. Did you watch the videos?
18. How did you feel about the dietary recommendations?
    a. What, if anything, was challenging about the dietary recommendations? At what point in the day or in what circumstances during the study did you have most trouble sticking to them?
    b. Did you set your own goals around the recommendations or change them in some way to make them more palatable or achievable?
    c. How can we better support your adoption of these types of dietary recommendations? *(Probe: videos about food, food prep, food shopping)*
19. Let's have a look at the My Prescription area of the website.
    a. How often, if at all, did you interact with this page?
        vii. For those who didn't interact with this page, why not?
    b. What, if anything, you do like about this page?
    c. What, if anything, would you change about this page?
        viii. *Probe about alternative formats (e.g., PDF, text messages, interactive app, etc.)*
    d. Is there any additional information that you would like to see on this page?
20. The website also contained a tool to report your diet and exercise progress.
    a. How often, if at all, did you use this feature?
        i. For those who didn't use the feature, why not?
    b. What, if anything, do you like about the process to track your progress?
    c. What, if anything, would you change about the process to track your progress?
    d. Which, if any, parts of your diet and exercise are you interested in tracking?
    e. Have you ever previously used a tool to track your diet or exercise? Which one(s)?
    f. Would you prefer to log diet/exercise somewhere else - such as using a paper diary?
21. There was also an area of the website called See Progress.
    a. How often, if at all, did you interact with this page?
        ix. For those who didn't interact with this page, why not?
    b. What, if anything, you do like about this page?
    c. What, if anything, would you change about this page?

   x. *Probe about alternative formats (e.g., PDF, text messages, interactive app, etc.)*

  d. Is there any additional information that you would like to see on this page?

VI. Future Directions (5 minutes; 80 minutes passed)

22. What do you think about patient websites where you can communicate with other people like you? If we added a feature where you could communicate anonymously with other study members (for example, within a forum on a specific topic), do you think you would use it? Why or why not?
23. [Levels 1-3 Only] If you were offered a 30 min diet or exercise phone counseling session, would you use it? If you could ask questions online to a diet or exercise coach at any point during the study, would you this feature?

VII. Wrap-up (5 minutes; 85 minutes passed)

24. Is there anything else you wanted to say in closing that you haven't yet had the opportunity to say?
25. You all have my email address, so don't hesitate to reach out if you have any additional thoughts that you'd like to share.

Thank you for your time and participation.

Focus Group Guide for Community of Wellness: Level 3

I. WELCOME AND INTRODUCTION (10 minutes; 10 minutes passed)
Welcome! Thank you all so much for coming. My name is Rebecca Graff and I will be the facilitator of this discussion group. We've invited you here to learn from you. Hearing about your experiences, ideas and opinions about the Community of Wellness study will help us to further develop materials and programs that will be useful to others who have gone through the same experiences as you, so we really appreciate you taking the time to come talk with us.

Before we begin, I'd like to walk through some key points about today's session:

A. Opinions:
The questions we will ask will be pretty straightforward. There are no right or wrong answers; we are interested in hearing what you think and what experiences you have had. All comments--both positive and negative--are needed. Please feel free to disagree with one another, but please don't criticize other people's opinions. Please also speak one at a time so that we can listen to your opinions.

B. Confidentiality:

- With your permission, we would like to record the conversation so that we can accurately capture all of your ideas.
- Taking part in this group is totally voluntary. You can leave at any time or not answer any question for any reason.
- All the information you provide will be kept confidential and will only be used by the people directly involved with this project. Your real names will not be used in any reports or publications about this research.
- If you need to refer to yourself in the third person, please only use your initials.
- Do not tell anyone outside the group what any particular person said in the group.

C. Housekeeping:

- Our discussion will last between 60 and 90 minutes.
- I really want to hear from each of you, so I hope that you will all feel comfortable voicing your opinions. Try to speak one at a time and be advised that I may call on particular people to ensure that we're hearing from everyone.
- I have some colleagues who will be listening in today. They're also interested in hearing your thoughts and feedback, but you won't be interacting with them directly.
- You can message the group using the "Chat" function or you can message an individual. To make sure that you know how to use the function, go ahead and send a message to

the group. Now send a message to me only. If you need to take a break or want to tell me anything confidentially, go ahead and send a direct chat to me.

- Can everyone see my screen?
- Are there any questions for me before we begin?

D. Introductions:

- Let's have you very briefly introduce yourselves and tell us where you're from. Please use your initials rather than your name. *[Suggest someone to begin.]*

II. Impressions of Study (10 minute; 20 minutes passed)

1. To begin, I'd like to hear about your experience with the Community of Wellness study. Overall, what were your impressions?
2. To what extent, if any, did you change your exercise or dietary habits during the study?
    a. How did you go about making the changes?
    b. What were the easiest changes for you to make? The hardest?
    c. What, if anything, made it difficult to make changes?
        i. Probe: Physical limitations, social support, cost, time, lack of knowledge of proper diet/exercise
    d. Since finishing the study do you feel that you have been able to sustain these changes? Why or why not? What would help you sustain healthy eating and exercise habits?
3. Which components of the study, if any, were the most effective in helping you to make diet or exercise changes?
    a. Did you feel that the study was missing anything that would have been especially helpful or would have motivated you more to make diet or exercise changes?
4. Did participation in the study ever feel time-consuming or burdensome? If yes, why?
5. What do you feel was the most important thing that you learned from participating in the study?

III. Overall Experience with Website (10 minutes; 30 minutes passed)

Now I'd like to discuss the website that was part of the study.

6. Did you access the website during the study? How often?
7. If you went to the website, did you access it on a computer, tablet or phone? What would your preferred platform be for future versions of the website?
8. What triggered you to use the website? (I.e., when did you use it?)
9. What did you like most about the website?
10. What did you like least or what was most difficult to use on the website?
    a. What is one thing you think we MUST change or do to improve this site?
11. Did you show the website to others or give a family member your login information?
    a. Do you think it would be useful to give family members logins? Why or why not?
    b. Should the website have a page with content tailored to family members or significant others? What kind of information do you think would be useful on such a page?
12. Is there anything else that you would have liked to have seen on the website?

   a. *Probe for: More support info about cancer treatment/management, blog posts, other types of exercise content*

IV. [Levels 3 and 4 Only] Technology (15 minutes; 45 minutes passed)

13. As part of the study, you should have received various text messages.
    a. What were your impressions of the text messages you received?
    b. How did you feel about the frequency with which you received text messages?
    c. What, if anything, you did you like about the text messages?
    d. What, if anything, would you change about the text messages?
        i. *Probe about alternative formats (e.g., PDF, website content, interactive app, etc.)*
    e. Let's talk about some of the specific types of text messages that you received. I'm curious to hear if you liked or disliked each of the following:
        i. quiz questions - questions that asked how many weekly servings of a certain food we recommend you eat
        ii. texts that gave an exercise suggestion and did not require a response
        iii. yes/no questions
        iv. texts with longer responses (such as questions that asked you what you have changed since starting the study)
    f. Is there any additional information or content that you would have liked to receive via text message? Perhaps that might have motivated you?
14. You also received a FitBit as part of the study,
    a. What were your impressions of the FitBit?
    b. How often, if at all, did you use the FitBit?
    c. What, if anything, you did you like about the FitBit?
    d. What, if anything, you did you dislike about the FitBit?
    e. How, it at all, did the Fitbit impact your behavior around exercise?
    f. How often, if at all, did you use FitBit area of the website?
        i. For those who didn't interact with this page, why not?
    g. What, if anything, you do like about this page?
    h. What, if anything, would you change about this page?
        ii. *Probe about alternative formats (e.g., PDF, text messages, interactive app, etc.)*
    i. Is there any additional information that you would like to see on this page?

V. [Levels 2-4 Only] Tailored Recommendations [Focus on Report Progress / See Progress] (20 minutes; 65 minutes passed)

As part of the study, you were provided with personalized exercise and diet recommendations.

15. How did you feel about the exercise recommendations?
    a. Did you follow the recommended exercise program? What was challenging about doing the exercise program?
    b. Did you read and use the safety information?
    c. Did you watch the videos?
16. How did you feel about the dietary recommendations?

a. What, if anything, was challenging about the dietary recommendations? At what point in the day or in what circumstances during the study did you have most trouble sticking to them?
b. Did you set your own goals around the recommendations or change them in some way to make them more palatable or achievable?
c. How can we better support your adoption of these types of dietary recommendations? *(Probe: videos about food, food prep, food shopping)*

17. Let's have a look at the My Prescription area of the website.
    a. How often, if at all, did you interact with this page?
        i. For those who didn't interact with this page, why not?
    b. What, if anything, you do like about this page?
    c. What, if anything, would you change about this page?
        ii. *Probe about alternative formats (e.g., PDF, text messages, interactive app, etc.)*
    d. Is there any additional information that you would like to see on this page?
18. The website also contained a tool to report your diet and exercise progress.
    a. How often, if at all, did you use this feature?
        i. For those who didn't use the feature, why not?
    b. What, if anything, do you like about the process to track your progress?
    c. What, if anything, would you change about the process to track your progress?
    d. Which, if any, parts of your diet and exercise are you interested in tracking?
    e. Have you ever previously used a tool to track your diet or exercise? Which one(s)?
    f. Would you prefer to log diet/exercise somewhere else - such as using a paper diary?
19. There was also an area of the website called See Progress.
    a. How often, if at all, did you interact with this page?
        iii. For those who didn't interact with this page, why not?
    b. What, if anything, you do like about this page?
    c. What, if anything, would you change about this page?
        iv. *Probe about alternative formats (e.g., PDF, text messages, interactive app, etc.)*
    d. Is there any additional information that you would like to see on this page?

VI. Future Directions (5 minutes; 70 minutes passed)

20. What do you think about patient websites where you can communicate with other people like you? If we added a feature where you could communicate anonymously with other study members (for example, within a forum on a specific topic), do you think you would use it? Why or why not?
21. [Levels 1-3 Only] If you were offered a 30 min diet or exercise phone counseling session, would you use it? If you could ask questions online to a diet or exercise coach at any point during the study, would you this feature?

VII. Wrap-up (5 minutes; 75 minutes passed)

22. Is there anything else you wanted to say in closing that you haven't yet had the opportunity to say?
23. Don't hesitate to reach out if you have any additional thoughts that you'd like to share. You all have my email address.

Thank you for your time and participation.

WILL INCORPORATE THE FOLLOWING IF TIME ALLOWS (PRIOR TO WRAP-UP)

VIII. Basic Website Components [For Level 3s, focus on Resources]
At this point, I'd like to bring ask you about some specific components of the website as I show them on my screen.

24. Currently we're looking at the dashboard page of the website.
    b. How often, if at all, did you interact with this page?
        i. For those who didn't interact with this page, why not?
    c. What, if anything, you do like about this page?
    d. What, if anything, would you change about this page?
        i. *Probe about alternative formats (e.g., PDF, text messages, interactive app, etc.)*
    e. Is there any additional information that you would like to see on this page?
25. Now let's move on to the Exercise Info page of the website.
    a. How often, if at all, did you interact with this page?
        i. For those who didn't interact with this page, why not?
    b. What, if anything, you do like about this page?
    c. What, if anything, would you change about this page?
        ii. *Probe about alternative formats (e.g., PDF, text messages, interactive app, etc.)*
    d. Is there any additional information that you would like to see on this page?
26. Now we're looking at the Diet Info page of the website.
    a. How often, if at all, did you interact with this page?
        iii. For those who didn't interact with this page, why not?
    b. What, if anything, you do like about this page?
    c. What, if anything, would you change about this page?
        iv. *Probe about alternative formats (e.g., PDF, text messages, interactive app, etc.)*
    d. Is there any additional information that you would like to see on this page?
27. Let's now jump to the Resources page of the website.
    a. How often, if at all, did you interact with this page?
        v. For those who didn't interact with this page, why not?
    b. What, if anything, you do like about this page?
    c. What, if anything, would you change about this page?
        vi. *Probe about alternative formats (e.g., PDF, text messages, interactive app, etc.)*
    d. Is there any additional information that you would like to see on this page?

e. For those who used the recipes, how did it go? Did you share w/ family member?
    i. Do you typically use recipes? How did our recipes compare?
    ii. *Probe: would it be better to give you one PDF recipe booklet that you could give to a partner or whoever cooked, if not yourself?*
    iii. For those who didn't use the recipes, why not? Generally don't use recipes, or were these recipes not appealing for some reason?
f. What about the shopping guides for each of the foods?
    i. Why/why not? How did it go?
    ii. Are the food decisions and cooking (shopping, choosing the menu, preparing food) shared by a partner? How can we support the shared shopping/cooking responsibilities within the website?

Focus Group Guide for Community of Wellness: Level 4

I. WELCOME AND INTRODUCTION (10 minutes; 10 minutes passed)

Welcome! Thank you all so much for coming. My name is Rebecca Graff and I will be the facilitator of this discussion group. We've invited you here to learn from you. Hearing about your experiences, ideas and opinions about the Community of Wellness study will help us to further develop materials and programs that will be useful to others who have gone through the same experiences as you, so we really appreciate you taking the time to come talk with us.

Before we begin, I'd like to walk through some key points about today's session:

A. Opinions:

The questions we will ask will be pretty straightforward. There are no right or wrong answers; we are interested in hearing what you think and what experiences you have had. All comments--both positive and negative--are needed. Please feel free to disagree with one another, but please don't criticize other people's opinions. Please also speak one at a time so that we can listen to your opinions.

B. Confidentiality:

- With your permission, we would like to record the conversation so that we can accurately capture all of your ideas.
- Taking part in this group is totally voluntary. You can leave at any time or not answer any question for any reason.
- All the information you provide will be kept confidential and will only be used by the people directly involved with this project. Your real names will not be used in any reports or publications about this research.
- If you need to refer to yourself in the third person, please only use your initials.
- Do not tell anyone outside the group what any particular person said in the group.

C. Housekeeping:

- Our discussion will last between 60 and 90 minutes.
- I really want to hear from each of you, so I hope that you will all feel comfortable voicing your opinions. Try to speak one at a time and be advised that I may call on particular people to ensure that we're hearing from everyone.
- I have some colleagues who will be listening in today. They're also interested in hearing your thoughts and feedback, but you won't be interacting with them directly.
- You can message the group using the "Chat" function or you can message an individual. To make sure that you know how to use the function, go ahead and send a message to the group. Now send a message to me only. If you need to take a break or want to tell me anything confidentially, go ahead and send a direct chat to me.
- Can everyone see my screen?
- Are there any questions for me before we begin?

D. Introductions:

- Let's have you very briefly introduce yourselves and tell us where you're from. Please use your initials rather than your name. *[Suggest someone to begin.]*

DI. **Impressions of Study** (10 minute; 20 minutes passed)

1. To begin, I'd like to hear about your experience with the Community of Wellness study. Overall, what were your impressions?
2. To what extent, if any, did you change your exercise or dietary habits during the study?
    a. How did you go about making the changes?
    b. What were the easiest changes for you to make? The hardest?
    c. What, if anything, made it difficult to make changes?
        i. Probe: Physical limitations, social support, cost, time, lack of knowledge of proper diet/exercise
    d. Since finishing the study do you feel that you have been able to sustain these changes? Why or why not? What would help you sustain healthy eating and exercise habits?
3. Which components of the study, if any, were the most effective in helping you to make diet or exercise changes?
    a. Did you feel that the study was missing anything that would have been especially helpful or would have motivated you more to make diet or exercise changes?
4. Did participation in the study ever feel time-consuming or burdensome? If yes, why?
5. What do you feel was the most important thing that you learned from participating in the study?

DII. **Overall Experience with Website** (10 minutes; 30 minutes passed)

Now I'd like to discuss the website that was part of the study.

6. Did you access the website during the study? How often?
7. If you went to the website, did you access it on a computer, tablet or phone? What would your preferred platform be for future versions of the website?
8. What triggered you to use the website? (I.e., when did you use it?)
9. What did you like most about the website?
10. What did you like least or what was most difficult to use on the website?
    a. What is one thing you think we MUST change or do to improve this site?
11. Did you show the website to others or give a family member your login information?
    a. Do you think it would be useful to give family members logins? Why or why not?
    b. Should the website have a page with content tailored to family members or significant others? What kind of information do you think would be useful on such a page?
12. Is there anything else that you would have liked to have seen on the website?
    a. Probe for: More support info about cancer treatment/management, blog posts, other types of exercise content

IV. [Level 4 Only] Coaching (20 minutes; 50 minutes passed)
As part of the study, you were given the opportunity to talk to diet and exercise coaches.

13. Did you talk to a diet coach?
    a. How helpful or unhelpful was talking to the diet coach?
    b. Did you get all of your questions answered?
    c. For those of you who didn't talk to a diet coach, why not?
    d. In your ideal world, how often would you choose to speak with a diet coach? How long should each call be?
14. Did you talk to an exercise coach?
    a. How helpful or unhelpful was talking to the exercise coach?
    b. Did you get all of your questions answered?
    c. For those of you who didn't talk to an exercise coach, why not?
    d. In your ideal world, how often would you choose to speak with an exercise coach? How long should each call be?
15. Let's have a look at the My Coach area of the website.
    a. How often, if at all, did you interact with this page?
        i. For those who didn't interact with this page, why not?
    b. What, if anything, you do like about this page?
    c. What, if anything, would you change about this page?
        ii. *Probe about alternative formats (e.g., PDF, text messages, interactive app, etc.)*
    d. Is there any additional information that you would like to see on this page?

V. [Levels 3 and 4 Only] Technology (15 minutes; 65 minutes passed)

16. As part of the study, you should have received various text messages.
    a. What were your impressions of the text messages you received?
    b. How did you feel about the frequency with which you received text messages?
    c. What, if anything, you did you like about the text messages?
    d. What, if anything, would you change about the text messages?
        i. *Probe about alternative formats (e.g., PDF, website content, interactive app, etc.)*
    e. Let's talk about some of the specific types of text messages that you received. I'm curious to hear if you liked or disliked each of the following:
        i. quiz questions - questions that asked how many weekly servings of a certain food we recommend you eat
        ii. texts that gave an exercise suggestion and did not require a response
        iii. yes/no questions
        iv. texts with longer responses (such as questions that asked you what you have changed since starting the study)
    f. Is there any additional information or content that you would have liked to receive via text message? Perhaps that might have motivated you?
17. You also received a FitBit as part of the study,
    a. What were your impressions of the FitBit?
    b. How often, if at all, did you use the FitBit?

    c. What, if anything, you did you like about the FitBit?
    d. What, if anything, you did you dislike about the FitBit?
    e. How, it at all, did the Fitbit impact your behavior around exercise?
    f. How often, if at all, did you use FitBit area of the website?
        i. For those who didn't interact with this page, why not?
    g. What, if anything, you do like about this page?
    h. What, if anything, would you change about this page?
        ii. *Probe about alternative formats (e.g., PDF, text messages, interactive app, etc.)*
    i. Is there any additional information that you would like to see on this page?

VI. Future Directions (10 minutes; 75 minutes passed)

18. How likely or unlikely would you be to use a website feature that allows you to track your diet and exercise? Which, if any, parts of your diet and exercise would be interested in tracking?
    a. Have you ever previously used a tool to track your diet or exercise? Which one(s)?
    b. Would you prefer to log diet/exercise somewhere else - such as using a paper diary?
19. What do you think about patient websites where you can communicate with other people like you? If we added a feature where you could communicate anonymously with other study members (for example, within a forum on a specific topic), do you think you would use it? Why or why not?
20. [Levels 1-3 Only] If you were offered a 30 min diet or exercise phone counseling session, would you use it? If you could ask questions online to a diet or exercise coach at any point during the study, would you this feature?

VII. Wrap-up (5 minutes; 80 minutes passed)

21. Is there anything else you wanted to say in closing that you haven't yet had the opportunity to say?
22. You all have my email address, so don't hesitate to reach out if you have any additional thoughts that you'd like to share.

Thank you for your time and participation.

WILL INCORPORATE THE FOLLOWING IF TIME ALLOWS (PRIOR TO WRAP-UP)

VIII. Basic Website Components [For Level 4s, focus on Resources]

At this point, I'd like to bring ask you about some specific components of the website as I show them on my screen.

23. Currently we're looking at the dashboard page of the website.
    a. How often, if at all, did you interact with this page?
        i. For those who didn't interact with this page, why not?
    b. What, if anything, you do like about this page?

c. What, if anything, would you change about this page?
    i. *Probe about alternative formats (e.g., PDF, text messages, interactive app, etc.)*
d. Is there any additional information that you would like to see on this page?

24. Now let's move on to the Exercise Info page of the website.
    a. How often, if at all, did you interact with this page?
        i. For those who didn't interact with this page, why not?
    b. What, if anything, you do like about this page?
    c. What, if anything, would you change about this page?
        ii. *Probe about alternative formats (e.g., PDF, text messages, interactive app, etc.)*
    d. Is there any additional information that you would like to see on this page?
25. Now we're looking at the Diet Info page of the website.
    a. How often, if at all, did you interact with this page?
        iii. For those who didn't interact with this page, why not?
    b. What, if anything, you do like about this page?
    c. What, if anything, would you change about this page?
        iv. *Probe about alternative formats (e.g., PDF, text messages, interactive app, etc.)*
    d. Is there any additional information that you would like to see on this page?
26. Let's now jump to the Resources page of the website.
    a. How often, if at all, did you interact with this page?
        v. For those who didn't interact with this page, why not?
    b. What, if anything, you do like about this page?
    c. What, if anything, would you change about this page?
        vi. *Probe about alternative formats (e.g., PDF, text messages, interactive app, etc.)*
    d. Is there any additional information that you would like to see on this page?
    e. For those who used the <u>recipes</u>, how did it go? Did you share w/ family member?
        i. Do you typically use recipes? How did our recipes compare?
        ii. *Probe: would it be better to give you one PDF recipe booklet that you could give to a partner or whoever cooked, if not yourself?*
        iii. For those who didn't use the recipes, why not? Generally don't use recipes, or were these recipes not appealing for some reason?
    f. What about the <u>shopping guides</u> for each of the foods?
        i. Why/why not? How did it go?
        ii. Are the food decisions and cooking (shopping, choosing the menu, preparing food) shared by a partner? How can we support the shared shopping/cooking responsibilities within the website?

IX. [Levels 2-4 Only] Tailored Recommendations [For Level 4s, focus on See Progress]

As part of the study, you were provided with personalized exercise and diet recommendations.

27. How did you feel about the exercise recommendations?

   a. Did you follow the recommended exercise program? What was challenging about doing the exercise program?
   b. Did you read and use the safety information?
   c. Did you watch the videos?
28. How did you feel about the dietary recommendations?
   a. What, if anything, was challenging about the dietary recommendations? At what point in the day or in what circumstances during the study did you have most trouble sticking to them?
   b. Did you set your own goals around the recommendations or change them in some way to make them more palatable or achievable?
   c. How can we better support your adoption of these types of dietary recommendations? *(Probe: videos about food, food prep, food shopping)*
29. Let's have a look at the My Prescription area of the website.
   a. How often, if at all, did you interact with this page?
      vii. For those who didn't interact with this page, why not?
   b. What, if anything, you do like about this page?
   c. What, if anything, would you change about this page?
      viii. *Probe about alternative formats (e.g., PDF, text messages, interactive app, etc.)*
   d. Is there any additional information that you would like to see on this page?
30. There was also an area of the website called See Progress.
   a. How often, if at all, did you interact with this page?
      ix. For those who didn't interact with this page, why not?
   b. What, if anything, you do like about this page?
   c. What, if anything, would you change about this page?
      x. *Probe about alternative formats (e.g., PDF, text messages, interactive app, etc.)*
   d. Is there any additional information that you would like to see on this page?
